# Effect of chromogranin A N-terminal fragment vasostatin-1 nano-carrier transfection on abdominal aortic aneurysm formation

**DOI:** 10.1080/21655979.2021.2005222

**Published:** 2021-11-29

**Authors:** Pingshan Wang, Wei Wang, Xingxing Peng, Fugui Ruan, Shiyao Yang

**Affiliations:** Department of Cardiovascular Surgery, Affiliated Hospital of Guilin Medical University, Guilin, Guangxi Province, China

**Keywords:** Chromogranin A, vasostatin-1, nano-carrier, abdominal aortic aneurysm, rats

## Abstract

The effects of transfection of N-terminal fragment of chromogranin A Vasostatin-1 (VS-1) nanocarriers on formation of abdominal aortic aneurysm (AAA) were discussed, and its mechanism was analyzed. Nanoparticles containing VS-1 genes were prepared by emulsion solvent evaporation method, and property of nanoparticles was examined. A total of 30 male SD rats were divided randomly into sham group (normal saline), AAA group (Type I porcine pancreatic elastase), and VS-1 group (Type I porcine pancreatic elastase+VS-1 suspension liquid). The diameter dilation of rats was measured, abdominal aortic morphology was observed by HE staining, and levels of AMP-activated protein kinase (AMPK) and mammalian target of rapamycin (mTOR) were examined by immunohistochemistry and Western blot. Correlation between AMPK as well as mTOR and diameter dilation was analyzed by Pearson correlation. VS-1 genes in VS-1 nanoparticles were 4.51% and coating efficiency of genes was 88%. Compared with rats in sham group, diameter dilation of rats in AAA group increased, damage of abdominal aorta in rats was obvious, p-AMPK decreased, and p-mTOR increased in AAA group. Compared with AAA group, diameter dilation of rats in VS-1 group decreased, abdominal aorta of rats was improved, p-AMPK increased, and p-mTOR decreased. The comparison of all above indicators had statistical meaning (*P* < 0.05). p-AMPK and p-mTOR were negatively (*r* = −0.9150 and *P* = 0.006) and positively correlated with the diameter dilation (*r* = −0.9206 and *P* = 0.001). VS-1 nanoparticles could inhibit the formation of AAA, which might be related to the activation of AMPK/mTOR signal path.

## 1. Introduction

Abdominal aortic aneurysm (AAA) is a common surgical pathological dilatation of the aorta. Most patients have no obvious symptoms before the tumor ruptures, but the prognosis of patients after the tumor ruptures is extremely poor with a fatality rate of 90% and above [1]. At present, the underlying pathogenesis of AAA is not fully understood. It is mainly believed that AAA is related to the degeneration of atherosclerotic aortic elastic media, the infiltration of inflammatory cells, the formation of new blood vessels, and the production and activation of various proteases and cytokines [2]. The main therapeutic method for AAA is surgical treatment, including classic open surgery and endovascular repair of the large or symptomatic aneurysms, while repair of small AAA does not bring obvious benefits [3]. Non-surgical treatment of AAA can reduce the risk of tumor rupture and of cardiovascular events. However, there is still no effective evidence for the value of drug interventions in curing AAA. Therefore, the research on the pathogenesis of AAA and the development of targeted therapeutic drugs play a critical role in promoting the treatment of AAA by therapeutic drugs.

N-terminal fragment of chromogranin A (CGA) Vasostatin-1 is 1–76 bit amino acid fragment of N-terminal of CGA. It is abundant in multiple types of cells, active in spectral antibacterial activity, protects body against infection, repairs endothelial cells, and regulates the homeostasis of cardiovascular system [4]. However, the application of VS-1 as a drug is a difficulty due to lack of an ideal carrier. With the growth of nanotechnology, the use of nanomaterials as carriers for gene-carrying drugs is a hot research topic [5]. The pCDNA3.1-VS-1 recombinant plasmid NP (referred to as VS-1 NP) prepared by the ESV has unique advantages in its structure and characteristics, but its regulation effect and mechanism on AAA have to be further studied. AMPK and mTOR are involved in proliferation, survival, metabolism, and other regulatory processes of cells [6]. Studies demonstrate that AMPK is involved in the occurrence and prevalence of AAA [7], but whether AMPK/mTOR SP is involved in the regulation of AAA by VS-1 remains to be further studied.

At present, there was few research on the formation of AAA by the transfection of nanocarriers. Based on the current situation, the nanoparticles containing VS-1 genes were prepared by emulsion solvent evaporation method in the research, and then the property of nanoparticles was identified and examined by polymerase chain reaction (PCR) amplification, DNA sequencing, and in vitro release curve. Meanwhile, the formation mechanism of AMPK/mTOR signal path in AAA was analyzed by the transfection of nanoparticle carriers into rat body experiment to lay the foundation for the gene treatment formed by further anti-AAA.

## 2. Materials and methods

### 2.1. Preparation of pCDNA3.1-VS-1 NP

**Figure 1.**
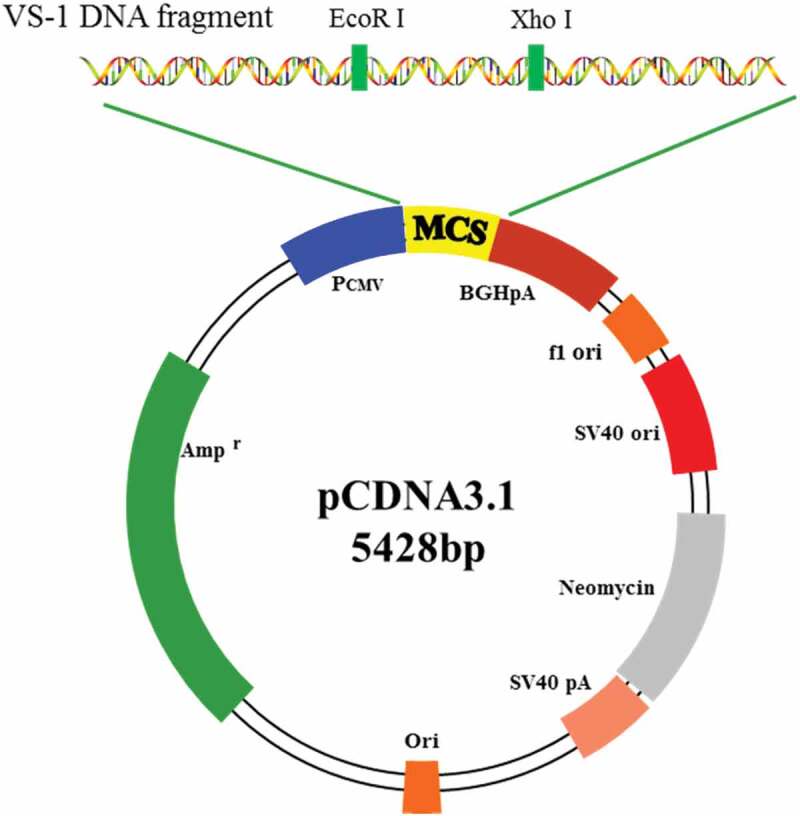
Schematic diagram for structure of pCDNA3.1-VS-1 NP

The coding sequence of the NTF VS-1 of CHGA and the multiple cloning sites on the pCDNA3.1 plasmid (Sangon Biotech (Shanghai) Co., Ltd.) were known based on Genebank. Three pairs of primers were designed, and EcoR I and Xho I were restriction enzyme digestion sites. The design and synthesis of primers were all completed by the Sangon Biotech (Shanghai) Co., Ltd. Target fragment of the primer was amplified in PCR with 31 cycles in total. After the digestion with EcoR I and Xho I, VS-1 DNA and pCDNA3.1 were purified according to the DNA gel purification kit (Takara), and the purified product was ligated. After that, the ligation product was transformed into competent *Escherichia coli* DH5α, which was placed in a 37°C incubator overnight after the Luria–Bertani (LB) medium was applied. Positive clones were screened and identified by restriction enzyme digestion and DNA sequencing. The structure of the constructed pCDNA3.1-VS-1 NP is shown in Figure 1.

### 2.2. Preparation of carrying pCDNA3.1-VS-1 NP

**Figure 2.**
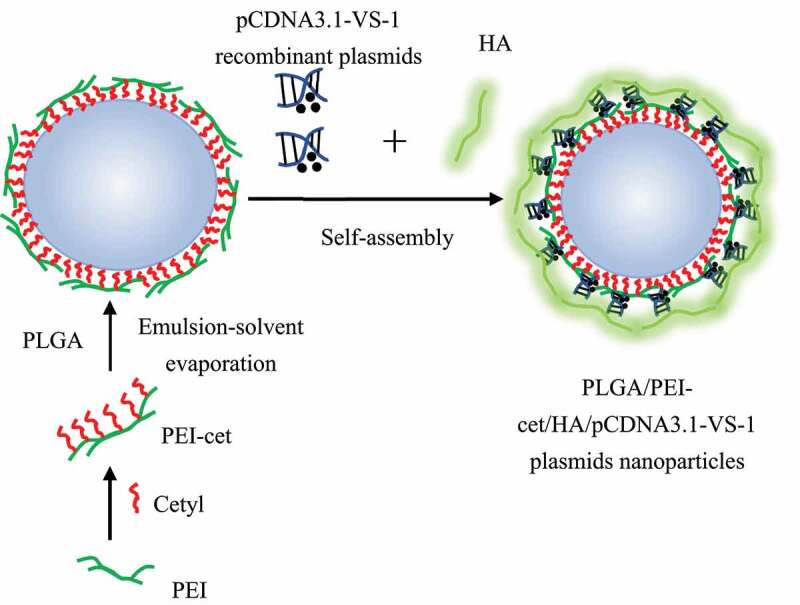
Preparation of carrying pCDNA3.1-VS-1 NP

Based on the study of Varshney et al. (2018) [8], the ESV method was used to prepare NPs containing the VS-1 gene. The schematic diagram of the preparation is shown in Figure 2. The specific steps were also illustrated. Preparation of PEI-cet: PEI-cet was synthesized by alkylation of hexadecyl bromide of polyethyleneimine (PEI) (PEI-cet) (25 kDa) [9]. Surface modification of polylactic acid – glycolic acid (PLGA) NP using PEI-cet: PLGA/PEI-cet NP was prepared according to the double ESV technology developed by Kumar et al. (2004) [10]; after being stirred overnight, PLGA was dissolved in dichloromethane (DCM) with a concentration of 10% (w/v); after being filtered with a 220 nm filter, 1 mL of DCM containing 12 mg of PEI-cet was added to 1 mL of PLGA solution, the mixed organic phase was poured into 20 mL 0.5% (w/v) water phase of polyvinyl alcohol (PVA) and stirred magnetically at 2,000 rpm at the temperature of 40°C to produce an oil-in-water emulsion to form a water/oil/water emulsion; then, it was stirred at the room temperature for more than 12 hours to evaporate the DCM; finally, the resulting microspheres were washed twice in deionized water, centrifuged at 16,000 g, and then freeze dried. Preparation of PLGA/PEI-cet/pCDNA3.1-VS-1 NP/HA complex: pCDNA3.1-VS-1 NP (0.1 mg/mL) and PLGA/PEI-cet NP solution (0.1 mg/mL) were mixed and allowed to automatically react in 150 mM phosphate buffer saline (PBS) buffer (pH 7.4), which was incubated at the room temperature for around 10 minutes; hyaluronic acid (HA) equaling to 1/3 of the weight of PEI-cet was poured into the mixed solution so that it could attach on the surface of NP, and then the final suspension was incubated at the room temperature for about 30 minutes with shaking to obtain VS-1 NP. Identification of VS-1 NP characteristics: the distribution and particle size of VS-1 NP were detected with a laser light scattering instrument; the surface morphology of VS-1 NP was observed with a scanning electron microscope (SEM). The accurately weighed VS-1 NP was extracted into the water phase, and the content of the VS-1 gene and the embedding rate were calculated. The DNA content in the release solution was measured in an in vitro gene release experiment, the results of which were illustrated by a cumulative release curve.

### 2.3. Experimental animals and grouping

A total of 30 male rats was purchased from Vital River Laboratories in Beijing. The age of these rats ranged from 8 to 12 weeks, and they weighed 180–250 g. All rats were kept in an animal room at 22 ± 1°C with light/dark (12/12) for cycle. They could drink and eat freely. All rats were grouped randomly into three groups: sham operation group (Sham), model group (AAA) and VS-1 group (VS-1) with 10 rats in each group. All the operations on rats in the research complied with *Animal Protection Law of People’s Republic of China*. Besides, the implementation of the research had been approved by Animal Ethics and Experimentation Committee.

### 2.4. Establishment and intervention measures of AAA model

The AAA model was established for the rats in model group and VS-1 group with reference to the methods proposed by Zhang et al. (2013) [11], which was slightly revised. Catheters were inserted into the cavity of abdominal aorta and 2 mL of type I porcine pancreatic elastase (25 U/mL) was perfused into the cavity. After 1 hour, the remaining liquid was discharged, and 2 mL of normal saline was perfused for sham operation group. After that, VS-1 nanoparticle suspension liquid was perfused into rats in VS-1 group through catheters, while normal saline was perfused into rats in model group. Suspension liquid remained in blood vessels for 30 min under the pressure of 202.65 kPa, and then catheters were removed, the incision was repaired, and iodophor was used for disinfection. From 1 day before operation to 1 day before the extraction of specimen during the second operation, rats were taken drug once every morning. All rats were raised for two weeks after operation, and the diameter of abdominal aorta was measured by vernier caliper before and after perfusion. Besides, dilatation of abdominal aortic diameter = abdominal aortic diameter after perfusion (mm)/abdominal aortic diameter before perfusion (mm) was calculated.

### 2.5. Calculation of incidence of AAA

The rats in each group were killed two weeks after the transfection of the above genes. The rats in each group were anesthetized to take out AA. After the diameter was measured, the artery was cut. The data was processed by EXECL2019 to calculate the maximum diameter. Then, part of the aorta was fixed with 10% of neutral formaldehyde for HE staining and immunohistochemistry. The other part was quickly frozen in liquid nitrogen and stored at −80°C for WB experiments.

### 2.6. HE staining

The AA were fixed with 4% neutral formaldehyde for 12 hours, dehydrated by alcohol at different levels, and cleared by xylene. The kidney tissue was embedded in paraffin, which was cut into 5 μm with a microtome, put into the water for unfolding, and then taken out with a glass slide for later use. After routine dewaxing and dehydration, the slices were stained with hematoxylin for 5 min, washed for 3 times, differentiated with 0.5% of hydrochloric acid ethanol for 7 s, washed again 3 times, and then stained with eosin for 1 min and washed 3 times. After dehydration with various levels of alcohol, they were cleaned and sealed with neutral gum film. The histological characteristics of the AA were observed under the Olympus optical microscope. Specific operation steps were taken based on the instructions of HE staining kit (Beijing Solarbio Science&Technology Co., Ltd.).

### 2.7. Immunohistochemical staining

**Figure 3.**
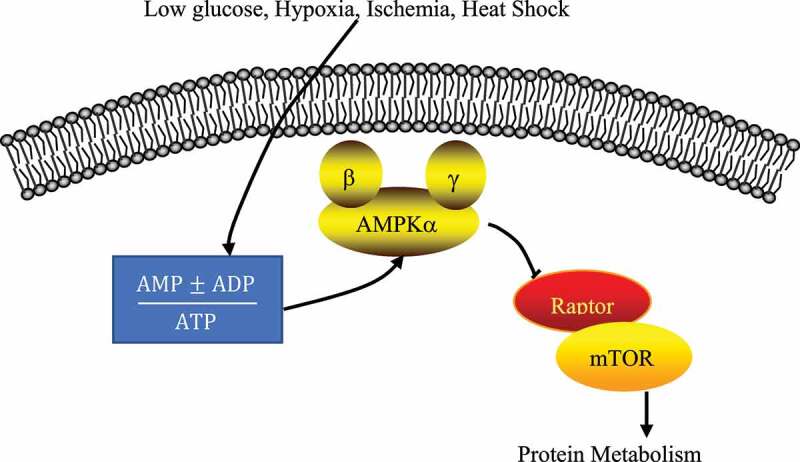
AMPK/mTOR SP

The involved AMPK/mTOR SP is shown in Figure 3, and the expressions of related proteins were detected according to this SP. After the above-mentioned slices were conventionally dewaxed and dehydrated, they were incubated with 3% of H_2_O_2_ for 10 min at the room temperature, washed 3 times, placed in a PBS solution, heated to 95°C for 10 min in a microwave, and then cooled. After blocked with 5% of fetal bovine serum for 45 min, it was added with p-AMPK primary antibody (1:50) or p-mTOR primary antibody (1:100), incubated overnight at 4°C, washed for 3 times, and added with secondary antibody dropwise to be incubated at the room temperature for 1 hour. After washing was repeated for 3 times, the slices were added with strept avidin biotin enzyme complex (SABC), incubated for 1 h at the room temperature, and added with diaminobenzidine (DAB) color solution, which should be removed 10 min later. The cell nucleus was lightly stained with hematoxylin and differentiated with 0.5% of hydrochloric acid ethanol for 7 s. Then, it was rinsed for 3 times and dehydrated with all levels of alcohol and clearing, and it was sealed with neutral gum. The primary antibody, secondary antibody, SABC, and DAB were all purchased from Beyotime Biotechnology (Shanghai, China). Expression of the target protein was observed under the Olympus optical microscope, and the positive expression rate was counted by the Image Pro Plus software. Specific operation steps were taken based on the instructions of immunohistochemistry kit immunohistochemistry kit (Shanghai Beyotime Biotechnology Co., Ltd).

### 2.8. WB

The radio immunoprecipitation assay (RIPA) protein lysate was adopted to extract the total protein (TP) in the AA, and the Bicinchoninic Acid (BCA) kit was utilized for quantitative analysis of TP. 20 μg of TP was added to the loading buffer, separated with 10% of sodium dodecyl sulfate polyacrylamide gel electrophoresis (SDS-PAGE), then transferred to the polyvinylidene fluoride (PVDF) membrane, and blocked with 5% of fetal bovine serum for 45 min to incubate the primary antibody; it was kept at 4°C overnight, and cleaned with Tris Buffered Saline Twee (TBST) for 3 times; after the secondary antibody was added, it was incubated for h at the room temperature, washed with TBST 3 times, and then electrochemiluminescence reagent (ECL) was utilized to emit light in a dark room. The target band was analyzed by using gel imaging system and Quantity One 4.6.2 of Bio-Rad (USA). Reduced glyceraldehyde-phosphate dehydrogenase (GAPDH) was adopted as an internal reference. Rabbit polyclonal antibodies p-AMPK and p-mTOR and goat anti-rabbit secondary antibodies were ordered from Beyotime Biotechnology (Shanghai, China).

### 2.9. Statistical analysis

All data were expressed as mean ± standard deviation, and statistical product and service solutions (SPSS) 22.0 software was adopted for statistical analysis. The comparison between the two groups was performed by *t* test, the comparison among multiple groups was analyzed by single-factor analysis of variance, and multiple comparisons were realized by LSD. Correlation between p-AMPK and p-mTOR PE to DE was analyzed by Pearson. *P* < 0.05 meant the difference was observable and significant statistically.

## 3. Results

Nanoparticles containing VS-1 genes were prepared by emulsion solvent evaporation method, and the property of nanocarriers was examined by SEM and in vitro release results in the research. The effects of nanocarriers on the formation of AAA was evaluated by the diameter dilation and the morphological changes of abdominal aortas of rats in each group in the research. In addition, the expressions of p-AMPK and p-mTOR in each group were analyzed by immunohistochemistry and Western blot method, and the correlation between p-AMPK as well as p-mTOR protein expression and diameter dilation was analyzed by Pearson correlation analysis, which aimed at constructing VS-1 nanoparticles successfully and identifying its influence in the formation of AAA.

### 3.1. Restriction enzyme identification of pCDNA3.1-VS-1 NP

**Figure 4.**
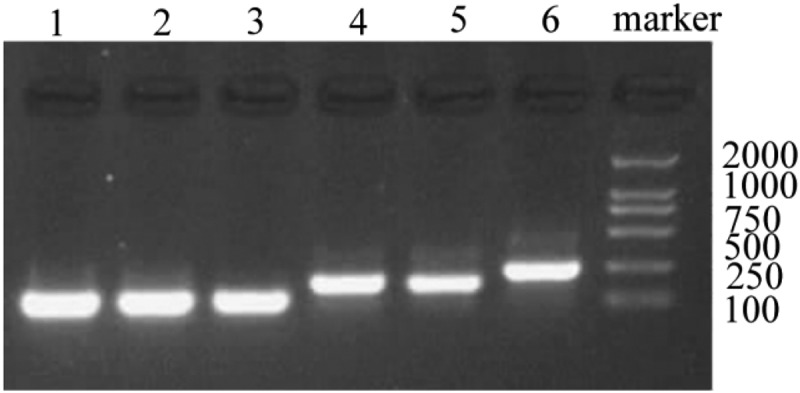
Amplification of VS-1 DNA with PCR

Note: each lane represented the VS-1 DNA fragment amplified from different primers. Lane 1 ~ 6 represented the primer 1, 2, 3, 1 + 2, 2 + 3, and 1 + 2 + 3, respectively.

Based on the designed primer 1, 2, and 3, specific VS-1 DNA fragments were amplified with PCR from primer 1, 2, 3, 1 + 2, 2 + 3, and 1 + 2 + 3. The amplified VS-1 DNA fragment was separated by 1% of agarose gel electrophoresis, whose result is displayed in Figure 4. The amplified VS-1 product was 228bp, which was consistent with the size mentioned in related literatures.

**Figure 5.**
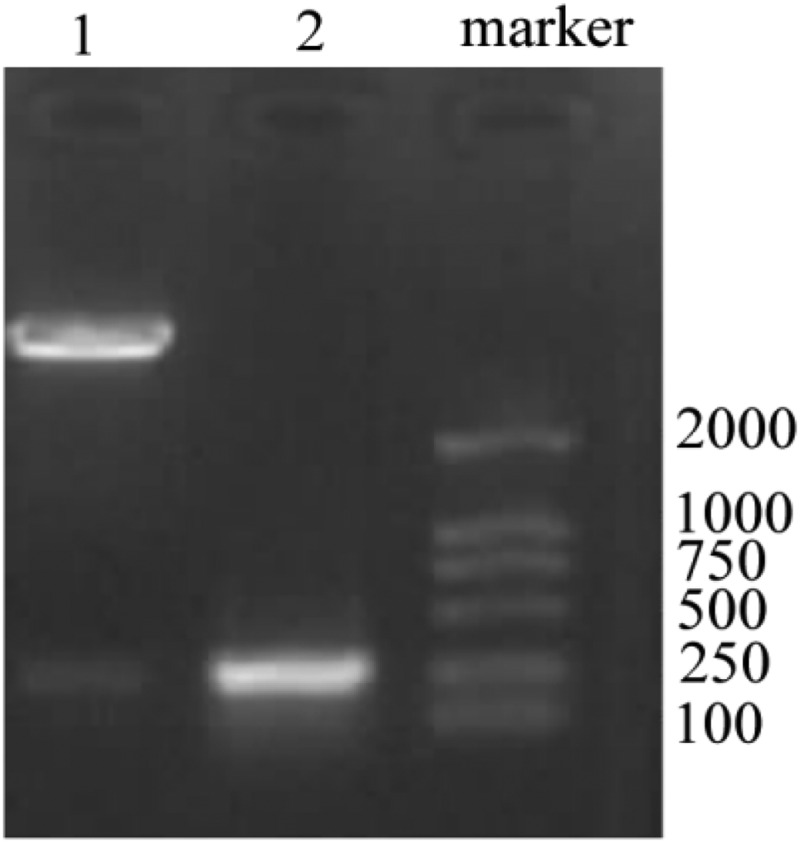
Identification results after digestion of pCDNA3.1-VS-1 NP

The positive clone plasmid DNA was extracted and screened, and digested by EcoR I+ Xho I to obtain a fragment of the same size as VS-1. The result is given in Figure 5. In lane 1, two strips were obtained after digestion. The lower-brightness band was the same size as the VS-1 gene in lane 1, which was the VS-1 fragment after digestion.

Lane 1: pCDNA3.1-VS-1 NP digested by EcoR I+ Xho I; lane 2: VS-1 gene.

### 3.2. DNA sequencing identification of pCDNA3.1-VS-1 NP

**Figure 6.**
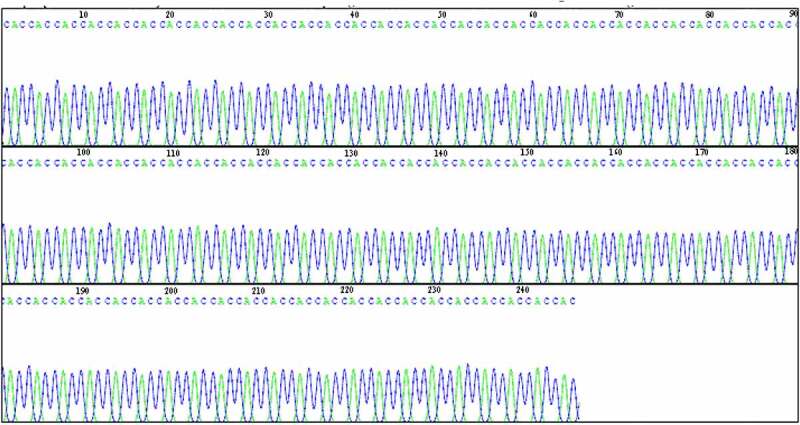
DNA sequencing of pCDNA3.1-VS-1 NP

The DNA fragments below lane 1 were tapped and purified for DNA sequencing. The sequencing results are shown in Figure 6, which indicated that the length of VS-1 on the constructed pCDNA3.1-VS-1 NP was 228bp (the first 1–17 bases were removed). The sequence was consistent with that reported result with 76 encoded amino acids, correct insertion sites and reading frames.

### 3.3. Extracorporeal characterizations of VS-1 NP

**Figure 7.**
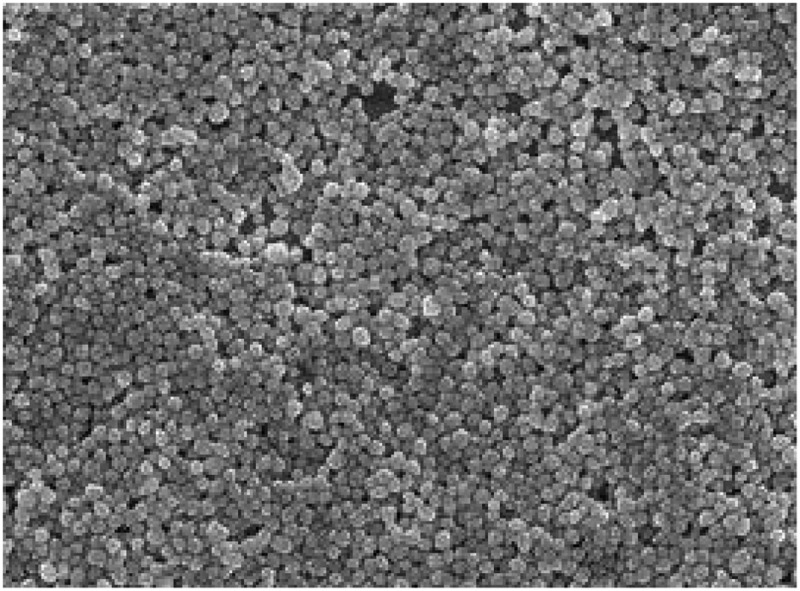
Microscopic morphologic surface of VS-1 NP

Note: ×15,000, and 20 kV.

The microscopic morphology results of VS-1 NP containing pCDNA3.1-VS-1 NP prepared by the ESV method are shown in Figure 7, which illustrated that the surface of the VS-1 NP was relatively smooth with round shape and uniform intact encapsulation.

**Figure 8.**
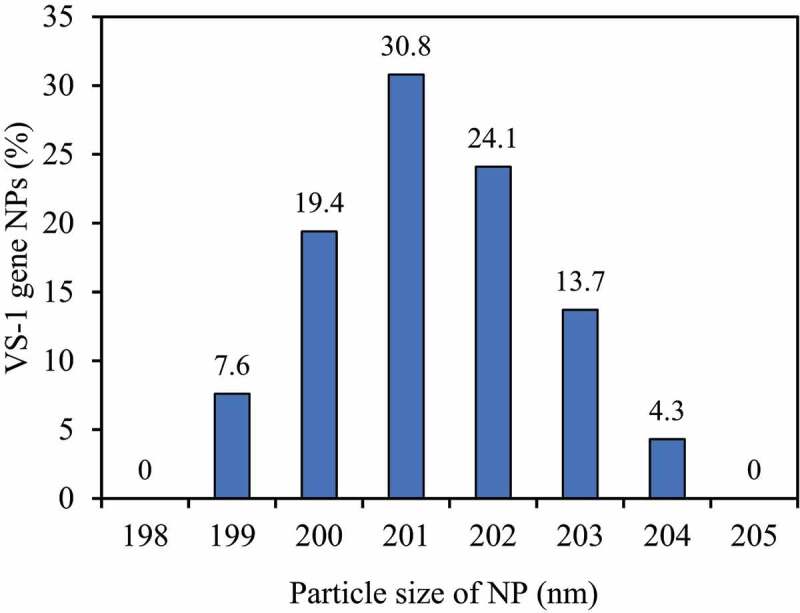
Curve for particle size distribution

The particle size distribution of VS-1 NP was shown in Figure 8. The particle size distribution range was narrow, mainly concentrated in 199–204 nm, with the average particle size of 201.9 nm, and the particle size distribution index was 0.066. In addition, the content of VS-1 gene in VS-1 NP was 4.51%, and the gene encapsulation efficiency was 88%.

**Figure 9.**
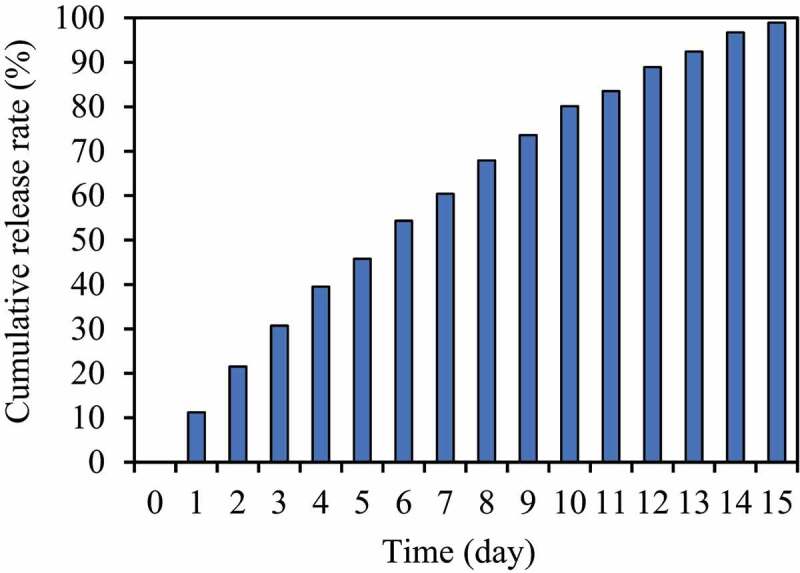
Cumulative release curve

The in vitro release curve of VS-1 NP was given below. Figure 9 illustrates that the in vitro release rate curve of VS-1 NP was close to a straight line in the first 0–6 days, which was in line with zero-order release. After about 7 days, the in vitro release rate began to slow down but kept rising slowly, and the stability of the release over past 14 days can be maintained generally.

### 3.4. Incidence of AAA and DE for rats in each group

**Figure 10.**
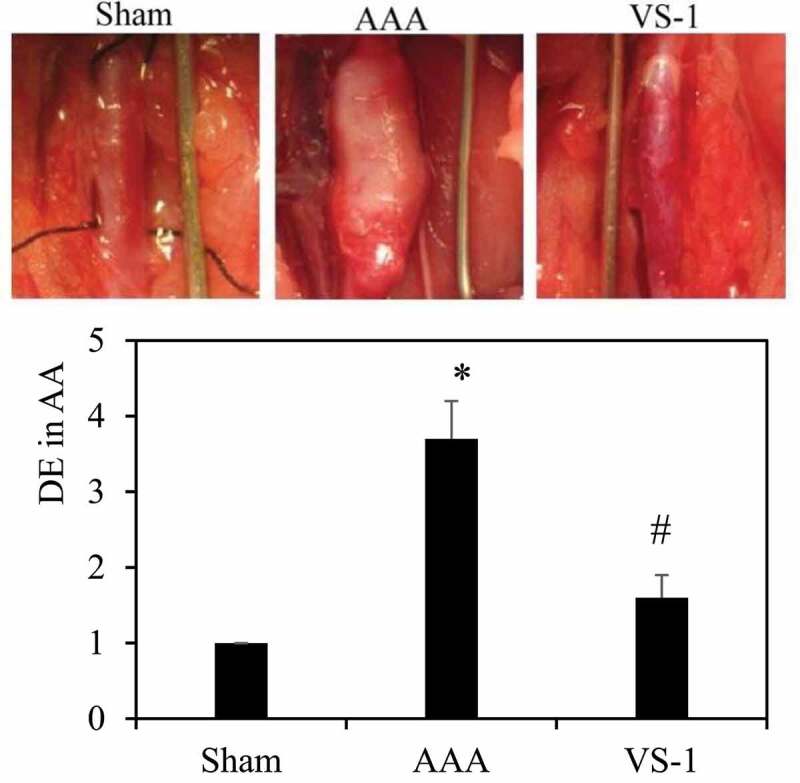
Incidence of AAA and DE for rats in each group

Note: (10 ×), * indicated *P* < 0.05 compared with sham; # indicated *P* < 0.05 compared with AAA.

VS-1 NP was transfected into the AA in rats of AAA model to observe its effect on the incidence of AAA. According to Figure 10, DE of AA greater than 2 indicated that the AAA model was successfully constructed, and the DE of AA in AAA were all greater than 2, which meant the model was constructed successfully in AAA. The DE of AA in AAA with transfected VS-1 NP was significantly smaller than that of the AAA. The results of statistical analysis revealed that the DE of rats in AAA increased dramatically compared with rats in sham (*P* < 0.05). Contrasted with the AAA, the DE of rats in the VS-1 was obviously reduced (*P* < 0.05). These results suggested that VS-1 NP could reduce AA expansion in AAA rats.

### 3.5. Morphological changes of AA in rats in each group

**Figure 11.**
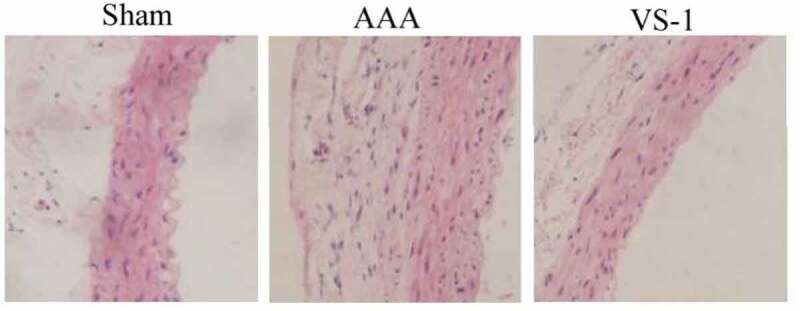
Comparison on HE staining results of AA for rats in each group (100 ×)

The morphological observation results of the AA are shown in Figure 11. The wall of AA for rats in sham was visible, and connective tissues, such as mesenchymal stem cells (MSCs), endothelial cells, and collagen fibers were neatly arranged. The AA wall in AAA suffered obvious damage, the three-layer membrane (intima, media, and adventitia) was unclear, elastic fibers were degraded, collagen fibers were proliferated, MSCs were shrunk and degenerated with irregular arrangement, and inflammatory cell infiltration could be observed. In the VS-1, the AA wall was damaged and repaired. The boundary of the three-layer membrane structure was clear, and the structure of MSCs and connective tissues was only slightly abnormal. These results indicated that VS-1 NP could repair the structural abnormalities of the AA caused by AAA.

### 3.6. Comparison on immunohistochemical results of AMPK and mTOR in each group of rats

**Figure 12.**
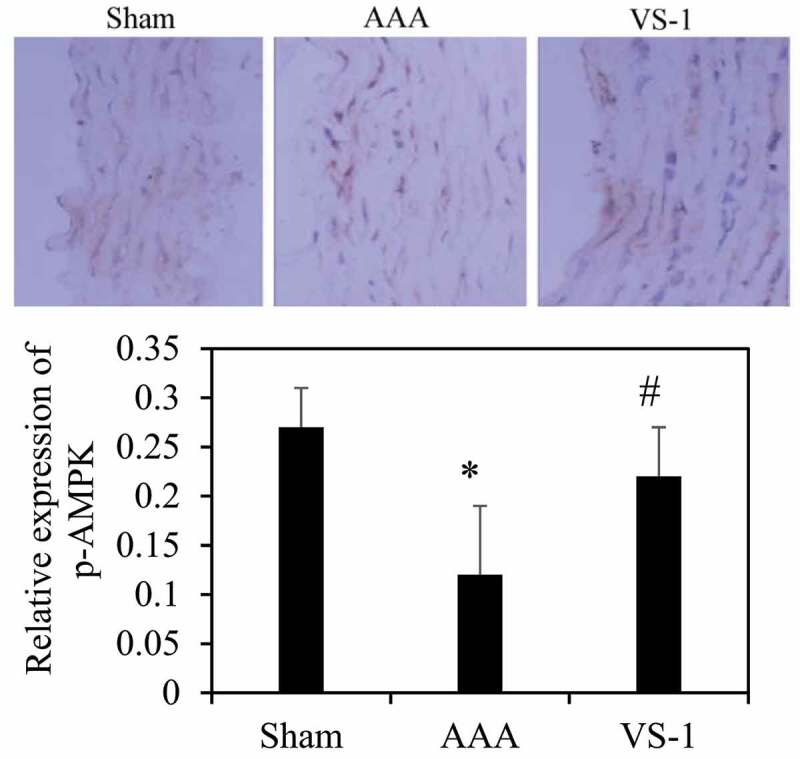
Comparison on immunohistochemical results of AMPK of rats in each group

Note: (10 ×), * indicated *P* < 0.05 contrasted with Sham; # indicated *P* < 0.05 compared to AAA.

**Figure 13.**
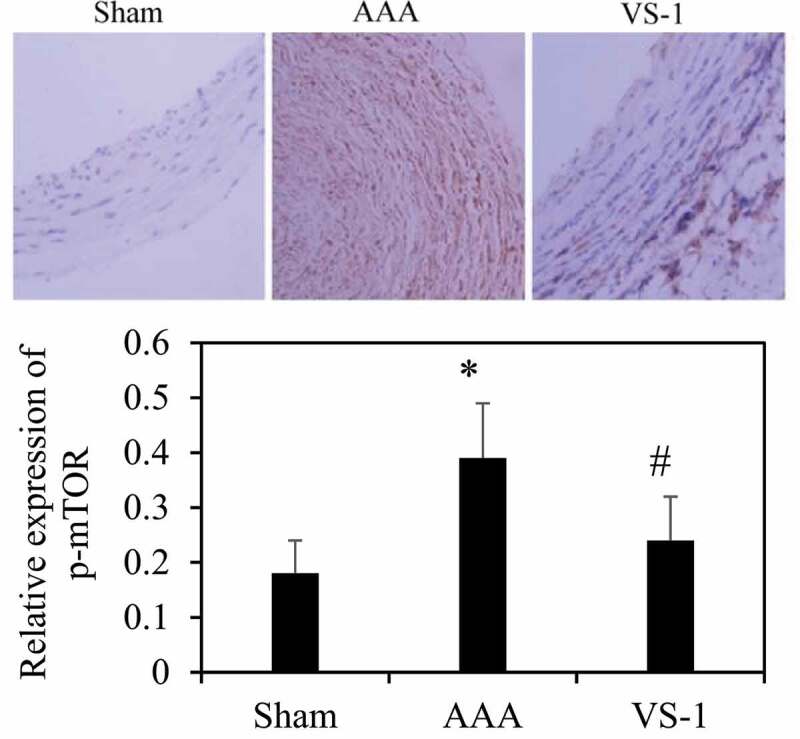
Comparison on immunohistochemical results of mTOR of rats in each group

Note: (100 ×), * indicated *P* < 0.05 contrasting with Sham; # indicated *P* < 0.05 in comparison to AAA.

To further explore the mechanism of VS-1 NP in improving AAA, the immunohistochemical results of p-AMPK and p-mTOR were analyzed, and the results are shown in Figures 12 and 13. Contrasted with the sham group, the p-AMPK protein in AAA group decreased remarkably (*P* < 0.05), while the PE of p-mTOR increased greatly (*P* < 0.05). In comparison to AAA group, the PE of p-AMPK in VS-1 group increased observably (*P* < 0.05), while the PE of p-mTOR decreased considerably (*P* < 0.05). These results indicated that the mechanism by which VS-1 NP improved AAA might be related to the activation of AMPK/mTOR SP.

### 3.7. Comparison of WB results of p-AMPK and p-mTOR in rats in all groups

**Figure 14.**
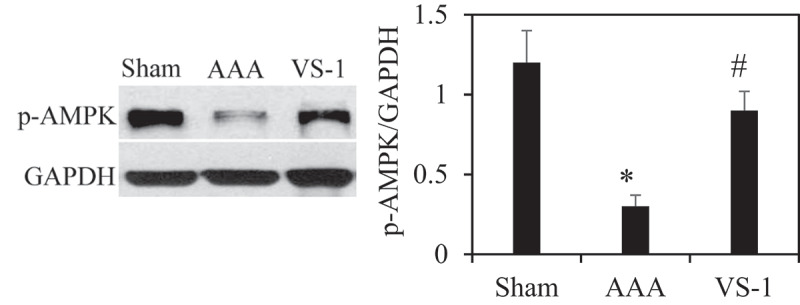
Comparison on p-AMPK PE in AA in each group

Note: * indicated *P* < 0.05 contrasted with Sham; # indicated *P* < 0.05 compared to AAA.

**Figure 15.**
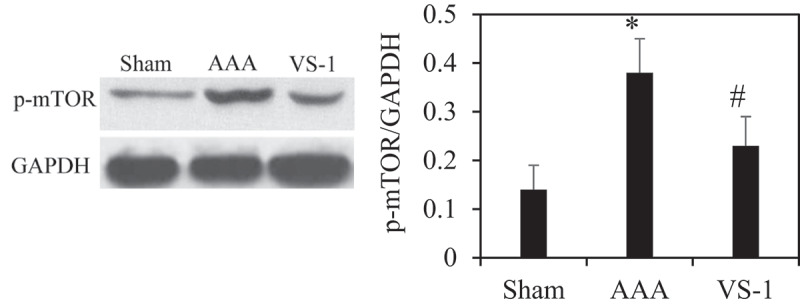
P-mTOR PE in AA in each group

Note: * indicated *P* < 0.05 compared to sham; # indicated *P* < 0.05 compared with AAA.

To further verify the role of AMPK/mTOR SP in the improvement of AAA by VS-1 NP, the WB results of p-AMPK and p-mTOR are illustrated in Figures 14 and 15. Comparatively speaking, the PE of p-AMPK in AAA group obviously decreased (*P* < 0.05), while the PE of p-mTOR was higher greatly than the sham group (*P* < 0.05). In comparison to AAA group, the p-AMPK PE in the VS-1 group increased remarkably (*P* < 0.05), while p-mTOR PE lower sharply (*P* < 0.05). These results further confirmed that the mechanism of VS-1 NP in improving AAA was related to the activation of AMPK/mTOR SP.

### 3.8. Analysis on correlation between DE and PE

**Figure 16.**
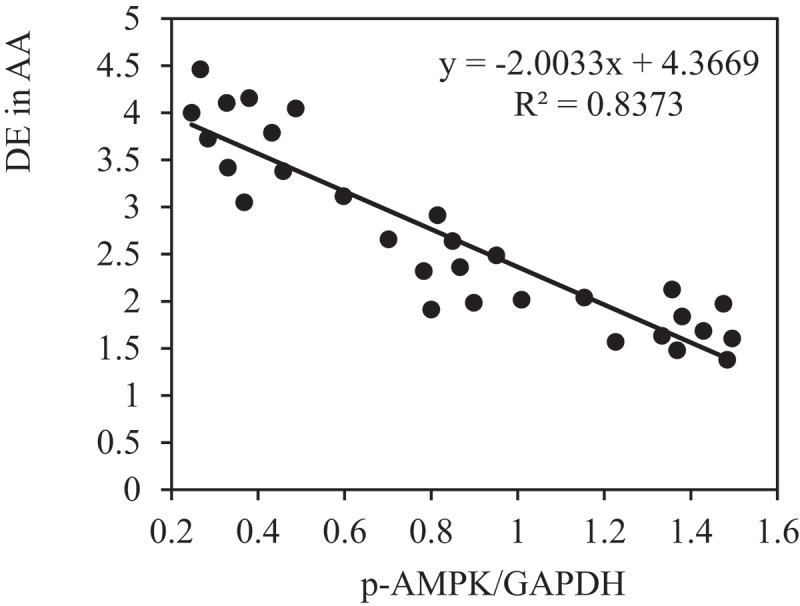
Correlation between p-AMPK PE and ED of rats in all groups

**Figure 17.**
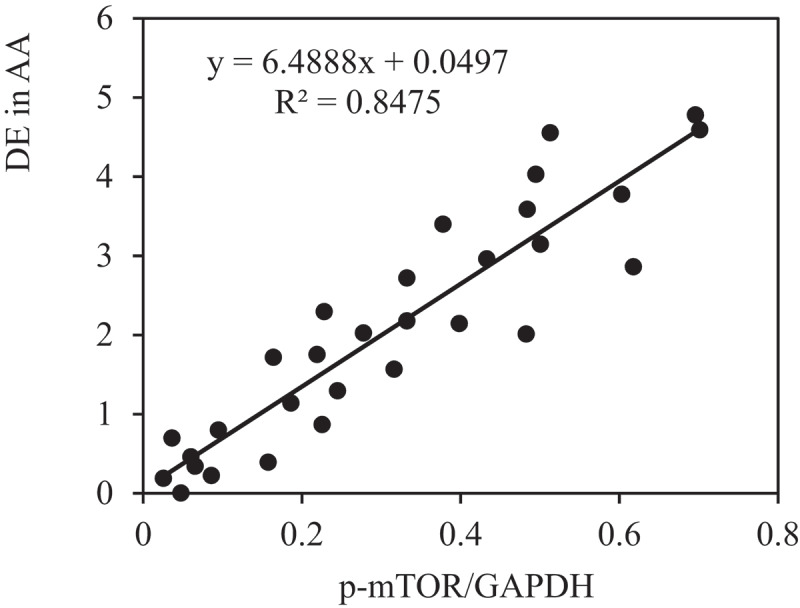
Correlation between p-mTOR PE and ED of rats in all groups

The correlation between the PE of p-AMPK and p-mTOR and DE in AA of rats in each group was analyzed to further verify the role of AMPK/mTOR SP in the improvement of AAA by VS-1 NP. Figures 16 and 17 show that p-AMPK PE was observably and negatively correlated with DE (*r* = −0.9150, and *P* = 0.006), and p-mTOR PE was remarkably and positively correlated with DE (*r* = 0.9206, and *P* = 0.001). These results suggested that AMPK/mTOR SP played a critical role in the improvement of AAA by VS-1 NP.

## 4. Discussion

Currently, universally applied gene vectors are classified mainly into viral vectors and non-viral vectors. Viral vector is applied in gene delivery with high transfection efficiency, but it has many disadvantages such as potential infectivity and immunogenicity, which limit its application. Non-viral vector has become an alternative strategy for gene delivery, such as hydrogels, micelles, and nanoparticles. Among various formulations, NP gene delivery systems have gradually attracted increasing attention and have been extensively studied due to their outstanding performance. In this study, the ESV method was utilized to prepare NP containing the VS-1 gene. SEM and in vitro release results showed that the surface of VS-1 NP was smooth and mostly round, and the particle size was concentrated at 199–204 nm. The average particle size was 201.9 nm, and the particle size distribution index was 0.066. In addition, the content of VS-1 gene in VS-1 NP was 4.51%, the gene encapsulation efficiency was 88%, and it could generally maintain stable release for more than 14 days. All these were consistent with the results of previous studies [12], indicating that the performance of the prepared VS-1 NP was reliable.

To further explore the effect of VS-1 NP on AAA, VS-1 NP was transfected into the AA of AAA model of rats to observe its effect on the incidence of AAA. The results showed that DE level of rats in AAA group obviously increased compared with that of rats in sham group. By comparison with AAA group, DE in the VS-1 group was greatly lower, suggesting that VS-1 NP could reduce the expansion of AA in AAA, which was consistent with the results of previous studies [13]. According to the structural analysis of VS-1 NP, it was speculated that the following reasons might be related to the repair of AAA by VS-1 NP. Firstly, HA in the outer layer of VS-1 NP could mediate endocytosis through boning with the receptor to promote the absorption of VS-1 NP into cells; secondly, hydrophilic HA might prevent the nanocomposite from interacting with serum components, thereby contributing to serum stability; finally, HA constituted the outermost layer of the sphere, which could prevent PEI from interacting with the cell membrane, thereby reducing the cytotoxicity of NP [14]. Therefore, VS-1 NP could be quickly and effectively absorbed by cells to play its role, and it had no toxic and side effects on cells, so VS-1 could exert an effective role in preventing AAA.

With the purpose of further exploring the effect of VS-1 NP on AAA, morphological changes of the AA of rats in each group were performed by HE staining. The results showed that AA wall of the rats in AAA group was damaged obviously, and the membrane structure was not clear and elastic, while elastic fiber degraded, collagen fiber proliferated, and MSCs shrunk and degenerated. In VS-1 group, the damage to AA wall of rats was eliminated, the membrane structure was clearly demarcated, and the MSCs and connective tissue structures were only slightly abnormal. It suggested that VS-1 NP could repair the structural abnormalities of the AA caused by AAA, which was similar to the results of Sato et al. (2018), which showed that VS-1 could inhibit the formation of atherosclerosis [15], and the deterioration of aortic elastic media caused by atherosclerosis was one of the main causes for AAA [16]. In this study, the elastic fibers in AAA group were degraded and the collagen fibers were proliferated, which were the manifestations of atherosclerosis. However, VS-1 could alleviate such symptoms, indicating that VS-1 NP could improve aortic sclerosis of AAA.

PEs of p-AMPK and p-mTOR of rats in each group were detected by immunohistochemistry and WB to further investigate the mechanism of VS-1 NP in improving AAA, and their correlations with DE in AA were analyzed. The results showed that the p-AMPK in AAA group greatly reduced PE than the shame and VS-1 group. In comparison with sham group, p-mTOR increased PE obviously, and PE in VS-1 group was lower than that in AAA group (*P* < 0.05). Correlation analysis revealed that p-AMPK PE was remarkably and negatively correlated with DE (*r* = −0.9150, and *P* = 0.006), and p-mTOR PE was positively and obviously related with the DE (*r* = 0.9206, and *P* = 0.001). Thus, it suggested that the AMPK/mTOR SP should be involved in the process of VS-1 NP to improve AAA, which was consistent with previous research results [17]. VS-1 could inhibit the activity of mTOR in small intestinal neuroendocrine tumor cells and further curb cell proliferation. Therefore, AMPK/mTOR SP should be involved in the improvement of AAA by VS-1 NP.

However, there are some defects in the research. For instance, the transfection of VS-1 nanoparticles is studied only by in vivo experiment. The signal path of AMPK/mTOR in the formation of AAA is not further explored further by in vitro experiment. These need to be further investigated in future research.

## 5. Conclusion

At present, the mechanism of action by VS-1 in inhibiting the formation of AAA was unknown. VS-1 nanoparticles were constructed successfully in the research, and its inhibition of the growth of AAA in rats was confirmed, which might be related to the activation of AMPK/mTOR signal path. The results provided a new idea for further treatment of the formation of AAA by genes.

## Data Availability

All data, models, and code generated or used during the study appear in the submitte.
